# Target and Non-target Site Mechanisms Developed by Glyphosate-Resistant Hairy beggarticks (*Bidens pilosa* L.) Populations from Mexico

**DOI:** 10.3389/fpls.2016.01492

**Published:** 2016-10-03

**Authors:** Ricardo Alcántara-de la Cruz, Pablo T. Fernández-Moreno, Carmen V. Ozuna, Antonia M. Rojano-Delgado, Hugo E. Cruz-Hipolito, José A. Domínguez-Valenzuela, Francisco Barro, Rafael De Prado

**Affiliations:** ^1^Department of Agricultural Chemistry and Edaphology, Campus of Rabanales, University of CordobaCordoba, Spain; ^2^Department of Agricultural Parasitology, Chapingo Autonomous UniversityTexcoco, Mexico; ^3^Institute for Sustainable Agriculture, Spanish National Research CouncilCordoba, Spain; ^4^Bayer CropScience MexicoMexico, Mexico

**Keywords:** 5-enolpyruvyl shikimate-3-phosphate synthase, *Bidens pilosa*, EPSPS2, resistance mechanisms, glyphosate translocation, TIPS mutation

## Abstract

In 2014 hairy beggarticks (*Bidens pilosa* L.) has been identified as being glyphosate-resistant in citrus orchards from Mexico. The target and non-target site mechanisms involved in the response to glyphosate of two resistant populations (R1 and R2) and one susceptible (S) were studied. Experiments of dose-response, shikimic acid accumulation, uptake-translocation, enzyme activity and 5-enolpyruvyl shikimate-3-phosphate synthase (EPSPS) gene sequencing were carried out in each population. The R1 and R2 populations were 20.4 and 2.8-fold less glyphosate sensitive, respectively, than the S population. The resistant populations showed a lesser shikimic acid accumulation than the S population. In the latter one, 24.9% of ^14^C-glyphosate was translocated to the roots at 96 h after treatment; in the R1 and R2 populations only 12.9 and 15.5%, respectively, was translocated. Qualitative results confirmed the reduced ^14^C-glyphosate translocation in the resistant populations. The EPSPS enzyme activity of the S population was 128.4 and 8.5-fold higher than the R1 and R2 populations of glyphosate-treated plants, respectively. A single (Pro-106-Ser), and a double (Thr-102-Ile followed by Pro-106-Ser) mutations were identified in the EPSPS2 gene conferred high resistance in R1 population. Target-site mutations associated with a reduced translocation were responsible for the higher glyphosate resistance in the R1 population. The low-intermediate resistance of the R2 population was mediated by reduced translocation. This is the first glyphosate resistance case confirmed in hairy beggarticks in the world.

## Introduction

Mexico is the top producer and exporter of limes and lemons worldwide (U.S. Department of Agriculture [USDA], 2016). Persian lime (*Citrus latifolia* Tan.) is the most economically important crop (Servicio de Información Agropecuaria y Pesquera [SIAP], 2016), because of its large volume of exports. Weeds are the main limiting factor in lime production, and the use of herbicides has been adopted as the main tool for weed control in this crop, mainly glyphosate, which is applied up to 4 treatments per year (Pérez-López et al., 2014). Glyphosate use has induced great changes in weed flora in Persian lime (*C. latifolia* Tan.) groves, where two cases of glyphosate resistance have been reported in Mexico: tropical sprangletop [*Leptochloa virgata* (L.) P. Beauv.], and hairy beggarticks (*Bidens pilosa* L.) (Pérez-López et al., 2014; Heap, 2016).

Hairy beggarticks is a native asteraceae from Mexico, widely spread over the country’s tropical and subtropical regions and in the world (Vibrans, 1995). It is an annual weed that reproduces itself by seeds, affecting annual and perennial crops (Rzedowski and Rzedowski, 2008). In 1991 it was reported as being resistant to paraquat in coffee plantations from Kenya, and in 1993 to ALS-inhibiting herbicides in soybean crops in Brazil (Heap, 2016). Hairy beggarticks is susceptible to glyphosate, but field prospections made by this research group in citrus orchard areas of Mexico have allowed the identification of glyphosate-resistant populations of this weed (Heap, 2016).

Glyphosate is a systemic non-selective herbicide that has been used globally for over 40 years in weed management (Duke and Powles, 2008; Szeìkaìcs and Darvas, 2012). When it is properly used, i.e., following label recommendations, it does not have any adverse effects on wildlife (Giesy et al., 2000). Glyphosate acts rapidly in reducing photosynthesis activity (Duke et al., 2003), and it is translocated with photosynthates from the leaves to the meristematic tissue to reach the target-site, achieving maximum uptake at 96 h after treatment (Cruz-Hipolito et al., 2011; González-Torralva et al., 2012b). Glyphosate is a phosphonomethyl derivative of the amino acid glycine (Szeìkaìcs and Darvas, 2012), and kills plants by preventing the synthesis of three essential amino acids (phenylalanine, tyrosine, and tryptophan; Franz et al., 1997), inhibiting the 5-enolpyruvyl shikimate-3-phosphate synthase (EPSPS; EC 2.5.1.19) (Duke and Powles, 2008; Szeìkaìcs and Darvas, 2012). Its effect is broader, the biosynthesis of chorismate, an intermediate in the shikimate pathway, is blocked causing the accumulation of high levels of shikimate-3-phosphate (Amrhein et al., 1980; Franz et al., 1997). Thereby, aromatic substances are disturbed in sensitive plants treated with this herbicide (Pline et al., 2002), affecting the production of flavonoids, phenolic compounds, monolignol polymerization, lignin synthesis, other secondary metabolites (Boerjan et al., 2003). This compounds can account for as much as 35% of a plant’s biomass (Franz et al., 1997). In addition, glyphosate causes a deregulation of the carbon flow to other essential pathways (Orcaray et al., 2012).

Depending almost exclusively on the use of glyphosate for weed management has led to the evolution of resistant populations (Alcántara-de la Cruz et al., 2016). During 20 years there was no evidence of any glyphosate-resistant weed populations (Duke and Powles, 2008). The first case reported was *Lolium rigidum* in 1996 (Powles et al., 1998). Since then, 258 glyphosate resistance cases have been reported in 35 weed species (Heap, 2016), mainly, but not only, influenced by adoption of transgenic glyphosate-resistant crops (Duke and Powles, 2008).

Glyphosate resistance in weeds is due to different mechanisms (Salas et al., 2015), grouped and commonly known as non-target site resistance (NTSR) and target site resistance (TSR) mechanisms (Sammons and Gaines, 2014). The NTSR mechanisms limit glyphosate reaching its site of action (EPSPS; Alcántara-de la Cruz et al., 2016). This group includes: reduced uptake (Michitte et al., 2007; de Carvalho et al., 2012), altered translocation (Perez-Jones et al., 2007), increased vacuolar sequestration (Ge et al., 2012), and metabolism to non-toxic compounds (de Carvalho et al., 2012; González-Torralva et al., 2012b), causing less glyphosate transport via phloem to the EPSPS. These mechanisms are influenced by enhanced physiological and biochemical characteristics (Alcántara-de la Cruz et al., 2016), and generally, each of these mechanisms confers moderate levels of glyphosate resistance (Yu et al., 2015).

The TSR mechanisms are those related to the EPSPS, either by a loss of affinity between the linking protein and glyphosate caused by mutations, or by the EPSPS overexpression (Sammons and Gaines, 2014). Different single mutations in the Pro-106 position (to Ala, Thr, and Leu) of EPSPS gene have been identified as conferring low-intermediate glyphosate resistance in weeds (de Carvalho et al., 2012; González-Torralva et al., 2012a, 2014; Alarcón-Reverte et al., 2015; Salas et al., 2015). Moreover, a double mutation was found in the Thr-102-Ile position followed by Pro-106-Ser conferring higher resistance in *Eleusine indica* (Chen et al., 2015; Yu et al., 2015). This double mutation is used in transgenic crops (Sammons and Gaines, 2014). Multiple EPSPS copy numbers and/or increased EPSPS expression are also involved in glyphosate resistance. These mechanisms have been described in mono and dicotyledonous weed species (Alarcón-Reverte et al., 2015; Chatham et al., 2015; Salas et al., 2015; Wiersma et al., 2015; Malone et al., 2016). In this paper, the target and non-target site mechanisms involved in glyphosate resistance of two resistant populations (R1 and R2) of hairy beggarticks in comparison to one susceptible (S) (as control), were studied by physiological, biochemical and molecular methods.

## Materials and Methods

### Biological Material and Experiment Conditions

Seeds of resistant populations (R1 and R2) were harvested directly in two Persian lime groves of the San Manuel farm, Puebla, Mexico, (20° 06′ 28″ N, 97° 09′ 34″ W) from at least 20 plants that had been survived to the last glyphosate treatment at the recommended field rate [720 g acid equivalent (ae) ha^-1^]. Persian lemon groves had a history of 6 (R2) and 13 (R1) years of continuous use of glyphosate (3–4 application per year). Seeds of a susceptible population (S) never treated were collected near the Persian lime groves. Seeds collected from a grove were bulked and constitute a sample from a single population.

Seeds were seeded on trays (15 cm × 15 cm × 8 cm) with peat saturated at a field capacity. The trays were covered with plastic layer until germination and placed in a growth chamber under controlled conditions (day/night temperature of 26/18°C, photoperiod of 16 h at 850 μmol^-2^s^-1^ of light intensity, and 60% relative humidity).

The seedlings were transplanted individually into 250 mL pots containing a mixture of sand/peat (1:1 v/v) + 0.4 g of fertilizer (NPK 17-09-11 + 2% MgO). The pots were placed in the growth chamber under the conditions described above and watered daily.

The glyphosate applications (Roundup Energy 45% w/v, Monsanto, Madrid, Spain) for the dose-response, foliar retention and shikimic acid assays were made with a Generation III Research Track Sprayer (DeVries Manufacturing Inc., Hollandale, MN, USA) equipped with an 8002EVS nozzle (TeeJet, Spraying System Spain, S.L., Madrid, Spain) delivering 200 L ha^-1^. The plants were treated with four true leaves counted from the bottom.

### Dose-Response Assays

Plants from the S, R1 and R2 populations were treated with the following doses of glyphosate: 0, 31.25, 62.5, 125, 250, 500, 1000, and 2000 g ae ha^-1^. At 21 days after treatment (DAT), the plants were cut off at ground level and wrapped in filter paper envelopes. Later, the plants were dried in a stove (JP Selecta S.A., Barcelona, Spain) at 60°C for 1 week and weighed to determine their dry weight. Data were expressed as a percentage of dry weight, compared to untreated control plants (Cruz-Hipolito et al., 2011). The experiment was arranged in a completely random design with 10 replications per dose. The assays were repeated twice.

### Foliar Retention Assays

The methodology adapted by González-Torralva et al. (2010) was employed. Na-fluorescein was used as labeling reagent for determination of herbicide solution amount was retained. Seven plants from each population, in a completely random design, were treated with a solution containing 360 g ae ha^-1^ of glyphosate (0.5 of field rate) + 100 mg L^-1^ Na-fluorescein. When the solution applied on the plant’s foliage dried (20–25 min after application), the treated plants were cut off at ground level and washed with 50 mL of NaOH 5 mM in a test tube shaking it vigorously for 30 s. The washing solution was recovered in glass flasks and the absorbance of fluorescein was immediately measured at 490_exc_/510_em_ nm (Hitachi F-2500 spectrofluorimeter). The plants were wrapped in filter paper envelopes and dried in a stove at 60°C for 1 week, and weighed. The retention was expressed in μL of glyphosate solution g^-1^ dry matter.

### Shikimic Acid Accumulation

An assay at different intervals of time was carried out. Plants from S, R1, and R2 populations, were treated with glyphosate at 360 g ae ha^-1^. Samples of 50 mg of tissue corresponding to the first and second leaf of treated and untreated plants (the latter used as a control) were cut at 24, 48, 72, and 96 hour after treatment (HAT). The samples were placed in an Eppendorf with 1 mL of HCl 1 M, immediately frozen in liquid nitrogen and stored at -40°C up to their analysis. The shikimic acid accumulation was determined by the methodology described by Cromartie and Polge (2002). Sample absorbance was measured with a spectrophotometer (Beckman DU-640, Fullerton, CA, USA) at 380 nm. The shikimic acid accumulation was obtained from the difference between treated and untreated plants, its rate was measured at between 24 and 96 HAT and the results were expressed in mg of shikimic acid g^-1^ fresh tissue. Five treated and untreated plants from each population at each time evaluated were used in a completely random design.

In leaf segment bioassay, young leaf disks 4 mm in diameter were taken until completing 50 mg of plant tissue from plants of hairy beggarticks populations S, R1, and R2 with four true leaves (Dayan et al., 2015). The disks were placed in Eppendorfs containing 999 μL of monoammonium phosphate (NH_4_H_2_PO_4_ 10 mM, pH 4.4). Next, 1 μL of glyphosate at different concentrations were added (0, 1, 10, 50, 100, 200, 400, 600, 1000, and 10000 μM). The samples were incubated for 24 h in the growth chamber under controlled conditions described above. Then the samples were frozen at -20°C until their analysis. After thawing the samples at room temperature, they were incubated at 60°C for 30 min. Volumes of 250 μL of HCl 1.25 N were added and incubated again at 60°C for 15 min. Aliquots of 250 μL were transferred to new Eppendorfs adding 500 μL of periodic acid (0.25% w/v) and sodium metaperiodate (0.25% w/v) at a ratio of 1:1. The samples were incubated at room temperature (25°C) for 90 min, and next, 500 μL of a mix of sodium hydroxide (NaOH 0.6 N) + sodium sulfite (Na_2_SO_3_ 0.22 N) was added at a ratio of 1:1, and mixed. Absorbance was measured at 380 nm in a spectrophotometer (Beckman DU-640). The experiment was arranged in a completely random design with three replications for each glyphosate concentration. The absorbance values were converted into mg of shikimic acid g^-1^ fresh weight.

### Uptake and Translocation of ^14^C-glyphosate

Plants from S, R1, and R2 populations were treated with a solution of ^14^C-glyphosate [glycine-2-^14^C] (specific activity 273.8 MBq mmol^-1^, American Radiolabeled Chemicals, Inc., Saint Louis, MO, USA) + commercial glyphosate. The solution applied contained a specific activity of 0.834 kBq^-1^ μL and a glyphosate concentration of 1.8 g ea L^-1^ (360 g ea ha^-1^ in 200 L). One drop of 1 μL plant^-1^ of solution was applied with a micropipette (Lab Mate HTL, Matosinhos, Portugal) on the adaxial surface of the first–second leaf. The treated leaf was washed three times separately with 1 mL of water-acetone (1:1 v/v) to recover the non-absorbed ^14^C-glyphosate at 24, 48, 72, and 96 HAT. The washing solution was mixed with 2 mL of scintillation liquid (Ultima Gold, Perkin-Elmer, BV BioScience Packard), and analyzed by liquid scintillation spectrometry (LSS) in a scintillation counter (LS 6500, Beckman Coulter Inc., Brea, Fullerton, USA). Complete plants were carefully removed from the pot and washed. They were divided into treated leaf, remainder of the plant and root, and stored individually in flexible combustion cones (Perkin-Elmer, BV BioScience Packard). The samples were dried in a stove at 60°C for 1 week and combusted in a biological oxidizer (Packard Tri Carb 307, Packard Instrument Co., Downers Grove, IL, USA). The CO_2_ released from the combustion was captured in 18 mL of a mix of Carbo-Sorb E and Permafluor (9:9 v/v) (Perkin-Elmer, BV BioScience Packard). The radioactivity of individual sample was quantified by LSS. The experiment was arranged in a completely random design with five replications per population at each time evaluated. The radioactive values were used to calculate recovery as: (kBq in treated leaf + kBq in plant + kBq in roots + kBq from washes/kBq total applied) × 100. The average total recovery of ^14^C-glyphosate applied was >94%.

The glyphosate translocation was visualized in plants from S, R1, and R2 populations. At 24, 48, 72, and 96 HAT, whole plants were washed, fixed on filter paper (25 cm × 12.5 cm) and dried at room temperature for 1 week. The samples were placed for 6 h beside a phosphor storage film (Storage Phosphor System: Cyclone, Perkin–Elmer Packard BioScience BV). A phosphor imager (Cyclon, Perkin-Elmer, Packard BioScience BV) was used to reveal the translocation. The experiment was carried out using three plants per population at each evaluation time.

### Glyphosate Metabolism

Randomized plants of the three hairy beggarticks populations were treated with 100 g ae ha^-1^. Untreated plants were used as controls. The methodology described by Rojano-Delgado et al. (2010) was used to determinate the percentage of glyphosate and its metabolites (aminomethyl phosphonate (AMPA), glyoxylate, sarcosine and formaldehyde) at 4, 8, and 12 DAT. Standard compounds used were provided by Sigma–Aldrich, Spain.

### Basal and Enzyme Activity of the EPSPS

Plants S, R1 and R2 populations were grown in pots (25 cm in diameter × 15 cm high: 4 plants per pot) under greenhouse conditions, in temperatures ranging from 17 to 31°C, and a photoperiod of 16 h. The natural light was complemented by 900 μmol^-2^s^-1^ photosynthetic photon flux density delivered by incandescent and fluorescent lights. The two youngest totally expanded leaves of plants with four true leaves were harvested until completing 5 g of foliar tissue for each population. Samples were frozen and stored at -40°C up to the protein extraction. The EPSPS extraction assays were conducted following the methodology described by Sammons et al. (2007). The total content of proteins in the raw extract was measured using the colorimetric method of Bradford (1976) following the manufacturer’s instructions with a Modified Lowry Kit for Protein Determination (Sigma–Aldrich, Madrid, Spain) following the manufacturer’s instructions.

The specific EPSPS activity in plants from S, R1, and R2 populations was studied in the presence and absence of glyphosate. In order to determine the EPSPS activity, a continuous assay of the release of inorganic phosphate was made with EnzChek Phosphate Assay Kit (Invitrogen, Carlsbad, CA, USA) following the manufacturer’s instructions. The glyphosate concentrations used were: 0, 1, 10, 100, 1000, and 10000 μM. Three replicates at each glyphosate concentration were analyzed. The release of phosphate above background level was measured during 10 min at 360 nm in a spectrophotometer (Beckman DU-640). The EPSPS activity was calculated to determine the amount of phosphate (μmol) released μg of total soluble protein (TSP)^-1^ min^-1^.

### Amplification and Sequencing of the EPSPS Gene

Samples (100–200 mg) of young leaf tissue were collected of plants from S, R1, and R2 populations and stored at -80°C for RNA extraction. The frozen samples were milled with liquid nitrogen in a STAR-BEATER 412-0167 mill (VWR International Eurolab S.L., Barcelona, Spain). Total RNA was isolated following the methodology described by Pistón (2013). Integrity of RNA was verified in 0.8% agarose gel and it was quantified by a NanoDrop ND-1000 spectrophotometer (Thermo Scientific, Walthman, MA, USA). First strand complementary DNA (cDNA) synthesis was carried out using 1 μg from the total RNA in all the samples. An iScript cDNA Synthesis Kit (Bio-Rad Laboratories, Inc., Hercules, CA, USA) was employed following the manufacturer’s instructions.

The PCR reactions were carried out with cDNA samples from each populations (R1, R2, S) using the following primers: Bidens-F13 (5′-TTGCCYGGRTCMAAGTCTTT-3′) and Bidens-R11 (5′-GTCCCAASTATCACTRTGTTC-3′) designed with software Primers3Plus^1^ based on EPSPS gene sequences of *Amaranthus tuberculatus* (Accession FJ869880.1, FJ869881.1), *A. palmeri* (FJ861242.1), *A. spinosus* (KF569213.1), *Conyza bonariensis* (EF200074.1), *C. canadensis* (AY545666.1, AY545667.1, FR872821.1), *C. sumatrensis* (AY834207.1), *Helianthus salicifolius* (AY545662.1) from the GenBank. A total volume of 25 μL which contained 50 ng of cDNA, 0.2 μM of each primer, 0.2 mM dNTP mix (PE Applied Biosystems; Life Technologies S.A., Madrid, Spain), 2 mM MgCl_2_, 1X buffer, and 0.625 units of a 100:1 enzyme mixture of non-proofreading (*Thermus thermophilus*) and proofreading (*Pyrococcus furiosus*) polymerases (BIOTOOLS, Madrid, Spain) per reaction using a thermocycler (Gene Amp PCR System 9700; Applied Biosystems, CA, USA). The PCR conditions were: 94°C for 5 min, 35 cycles of 94°C for 30 s, 55°C for 30 s, and 72°C for 1 min; and a final extension at 72°C for 10 min. PCR products were checked by 1% agarose gel. The amplified fragments of 639 bp in length included the Thr-102 and Pro-106 positions, which corresponds to the sequence of the EPSPS gene of *Arabidopsis thaliana* (GenBank: CAA29828.1), point mutations associated with glyphosate resistance in weeds (Sammons and Gaines, 2014; Chen et al., 2015; Yu et al., 2015).

The PCR products were ligated using the pGEM-T Easy Vector System (Promega Biotech Ibérica, SL, Madrid, Spain) following the manufacturer’s instructions, and cloned into competent cells of *Escherichia coli* DH5α. Positive transformants were selected. The fragment insertion was confirmed through a PCR using the M13F (5′-CGCCAGGGTTTTCCCAGTCACGAC-3′) and M13R (5′-TCACACAGGAAACAGCTATGAC-3′) primers at a total volume of 15 μL containing 0.2 μM of each primer, 0.2 mM dNTP mix (PE Applied Biosystems; Life Technologies S.A., Madrid, Spain), 2 mM MgCl_2_, 1X buffer, and 0.625 units of non-proofreading (*Thermus thermophilus*) polymerase (BIOTOOLS, Madrid, Spain) per reaction. The PCR conditions were as follows: 94°C for 5 min, 28 cycles of 94°C for 30 s, 50°C for 30 s, and 72°C for 1 min; and a final extension at 72°C for 7 min. The plasmids were purified with the illustra plasmidPrep Mini Spin kit (GE Healthcare, Buckinghamshire, UK), following the manufacturer’s instructions. Sanger sequencing was carried out by the STABVIDA sequencing service (Caparica, Portugal). Five biological samples were used per population. A total of 15 clones from each population were sequenced. The assembly of the sequences was carried out by SeqMan Pro (Version 11, DNASTAR; Wisconsin, USA) and Geneious (Version 8.1.8, Biomatters Ltd, Auckland, New Zealand) software’s.

A second EPSPS sequencing with 15 new individuals from R1 population to confirm mutations was carried out. A total of 45 clones were sequenced.

The hairy beggarticks EPSPS cDNA sequences information can be found in GenBank with accession numbers KU984452–KU984458.

### Statistical Analysis

The dry weight and survival percentage data were submitted to a non-linear regression analysis. The dose needed to reduce the growth of a population by 50% (ED_50_), the mortality by 50% (LD_50_), and to inhibit EPSPS activity by 50% (*I*_50_) were calculated. The log-logistic model was conducted using SigmaPlot (Version 11.0, Systat Software, Inc., USA) software. The statistical model is:

$$\begin{array}{c} Y=c+\{(d-c)/[1+{\left(x/g\right)}^{b}]\} \end{array}$$

Where *Y* is the dry weight, survival and/or EPSPS inhibiting percentage with respect to the untreated control, *c* and *d* are coefficients corresponding to the upper (maximum growth) and lower (minimum growth) asymptotic limits), *b* is the Hill slope, *g* is the herbicide dose (ED_50_, LD_50_ or *I*_50_) at the mean point of inflection between the upper and lower asymptote and *x* (independent variable) corresponds to the herbicide dose.

Statistical analyses between the hairy beggarticks populations were performed using Statistix version 8.0 Analytical Software. The experimental results were subjected to analysis of variance, and means were compared using Tukey’s or LSD test’s at the 95% probability level.

## Results

### Dose-Response

**FIGURE 1:**
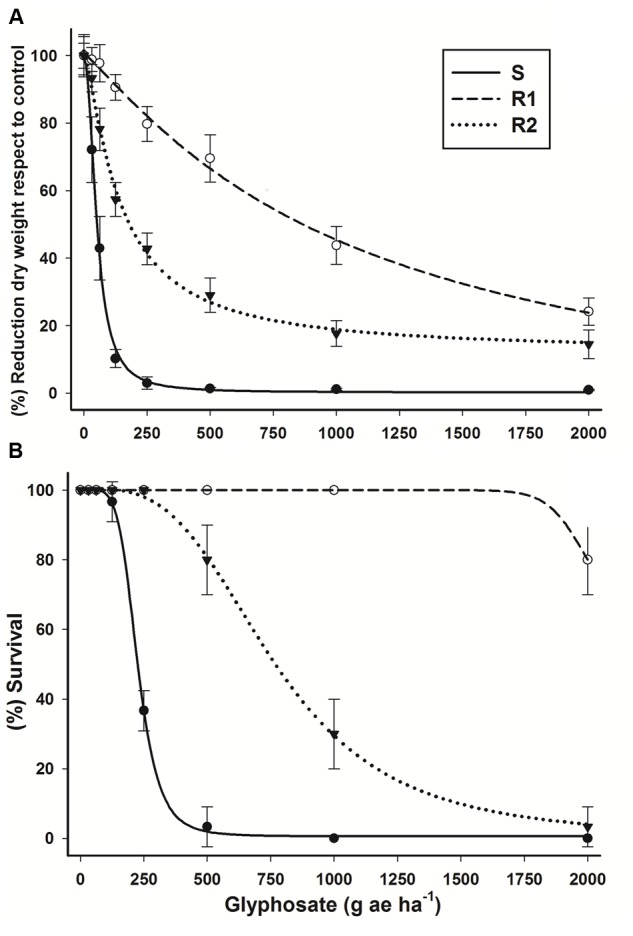
**Log–logistic curves of glyphosate-susceptible and -resistant *Bidens pilosa* populations evaluated at 21 days after treatment (DAT). (A)** Dose-response curve with respect to percentage of dry mass reduction. The equations of log–logistic curves to estimates the ED_50_ values are: S: *Y* = 0.178 + {(99.354 - 0.178)/[1 + (dose/ED_50_)^2.134^]}; R1: *Y* = -3.295 + {(100.56 + 3.295)/[1 + (dose/ED_50_)^1.140^]}; R2: *Y* = 12.121 + {(101.39 - 12.121)/[1 + (dose/ED_50_)^1.288^]}. **(B)** Dose-response curve with respect to percentage of survival. The equations of log–logistic curves to estimates the LD_50_ values are: S: *Y* = 0.623 + {(100.17 - 0.623)/[1 + (dose/LD_50_)^5.396^]}; R1: rates used did not permit to estimate LD_50_ value; R2: *Y* = -1.156 + {(101.47 + 1.156)/[1 + (dose/LD_50_)^3.238^]}. Vertical bars represent the standard error of the mean (*n* = 10).

This experiment confirmed the resistance of R1 and R2 hairy beggarticks populations to glyphosate. A large reduction of biomass in population S was observed at low glyphosate doses in comparison to that of the resistant populations (**Figure 1A**). The ED_50_ value for the S population was 51.7 g ae ha^-1^, whereas the R1 and R2 populations exhibited a higher ED_50_, with resistance index (RI) values (ED_50_R/ED_50_S) of 20.4- and 2.7-fold more resistant, respectively (**Table 1**). The R1 population showed an ED_50_ value 1.46-fold higher than the glyphosate field rate recommended (720 g ae ha^-1^).

**Table 1:** ED_50_, LD_50_, and ID_50_ values of glyphosate-susceptible and -resistant *Bidens pilosa* populations.

Population	ED_50_ (g ae ha^-1^)	RI*^a^*	LD_50_ (g ae ha^-1^)	RI*^a^*	*I*_50_ (μM)	RI*^a^*
S	51.7 ± 2.3		225.4 ± 4.4		0.96 ± 0.0	
R1	1055.8 ± 34.9	20.4	>2000	>8.7	122.7 ± 2.1	128.4
R2	142.7 ± 10.8	2.8	774.4 ± 79.5	3.4	8.2 ± 1.1	8.5

**^a^*RI, Resistance indexes (R/S) calculated using the ED_50_, LD_50_, or *I*_50_ values of the resistant populations respect to the susceptible population. ±Standard error of the mean.*

According to LD_50_ values, R1 and R2 populations were 9.5- and 3.4-fold more resistant than the S population (**Figure 1B**; **Table 1**). A field rate of glyphosate showed total control for the S population. A glyphosate field rate > 2.7-fold was needed to kill 50% R1 population plants. Even though the R2 population presented fivefold less ED_50_ than the field rate, just 50% of the population was eradicated (LD_50_ = 774.4 g ae ha^-1^) with this rate. The chlorosis symptoms caused by glyphosate application in resistant populations became evident as the glyphosate doses increased, although they were not sufficient to control the R1 population, in which plants survived treatment at 21 DAT, and continued growing up to the reproductive phase.

### Foliar Retention

There were significant differences in foliar retention between hairy beggarticks populations (*P* = 0.0045; DF = 2; *n* = 21). The R2 (A) population retained the highest amount of glyphosate solution (392 ± 39 μL g^-1^ of dry weight), followed by the S (B) population with a mean value of 343 ± 34 μL g^-1^ of dry weight, whereas the R1 (B) population reached a mean of 328 ± 32 μL.

### Shikimic Acid Accumulation

**FIGURE 2:**
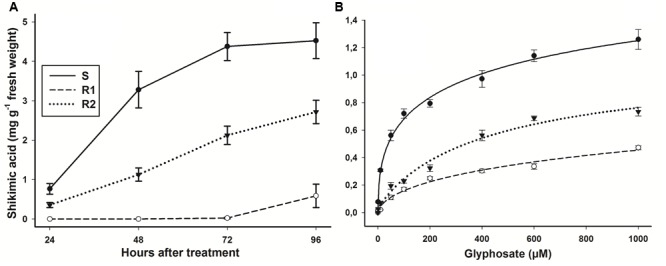
**Shikimic acid accumulation of glyphosate-susceptible and -resistant *B. pilosa* populations. (A)** Shikimic acid accumulation after a glyphosate application at 360 g ae ha^-1^ at different intervals of time. **(B)** Shikimic acid accumulation at different glyphosate concentrations. Vertical bars represent the standard error of the mean (*n* = 6 technical replicates).

In the assay at different time intervals with whole plants, the S population presented an accumulation of 0.76 ± 0.13 mg shikimic acid g^-1^ of fresh weight at 24 HAT, reaching up to 4.5 ± 0.52 mg g^-1^ of fresh weight at 96 HAT (**Figure 2A**). The S and R2 populations showed an accumulation of shikimic acid since 24 HAT, while R1 population alone presented a considerable accumulation as from 72 HAT. Thus, S population was 7.7-fold more susceptible than population R1, and 1.6-fold in comparison to R2 population. The leaf segment bioassay results obtained from different glyphosate concentrations were consistent with the results obtained in the assays with the whole plants. The hairy beggarticks populations accumulated shikimic acid as the glyphosate concentrations increased (**Figure 2B**). The greater accumulation of shikimic acid exhibited by population S was consistent with the greater reduction in growth observed in plants of these populations at these low rates (**Figure 1A**). Populations R1 and R2 were 3.3- and 1.9-fold more resistant, respectively, than S population.

### ^14^C-glyphosate Uptake and Translocation Assays

**FIGURE 3:**
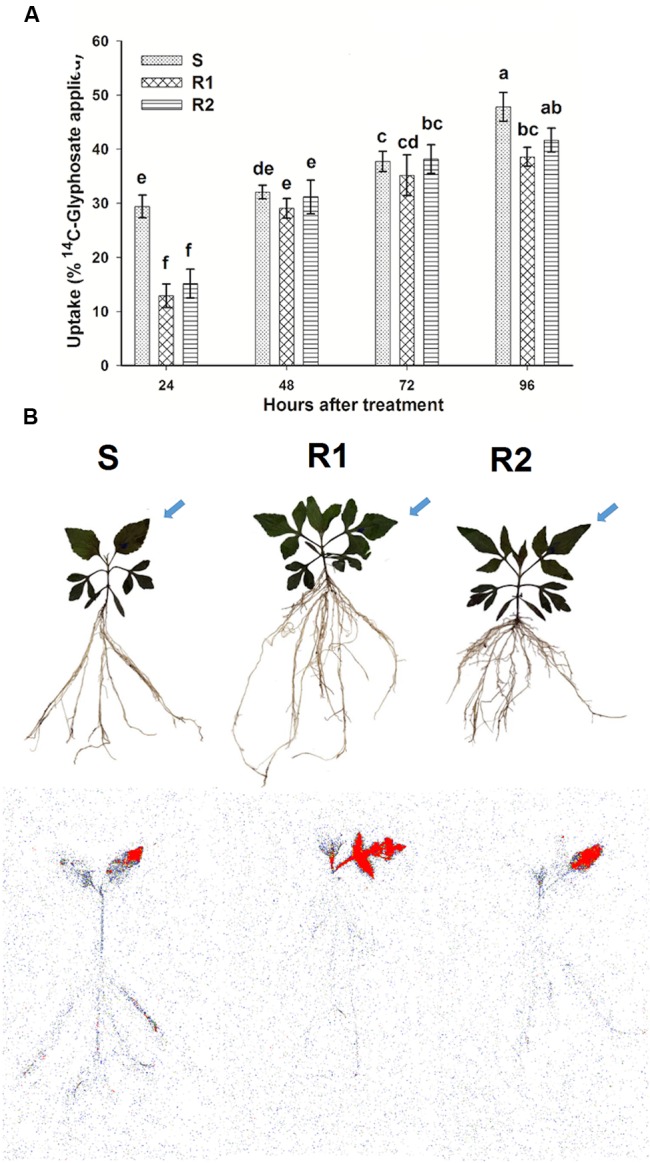
**^14^C-Glyphosate uptake and translocation in glyphosate-susceptible and -resistant plants of *B. pilosa* populations. (A)** ^14^C-glyphosate uptake in glyphosate-susceptible and -resistant *B. pilosa* plants. Different letters are statistically different at 95% probability determined by LSD test. Vertical bars represent the standard error of the mean (*n* = 5). **(B)** Digital images (upper plants) and autoradiograph images (lower plants) that show the distribution of ^14^C-glyphosate translocation in glyphosate-susceptible and -resistant *B. pilosa* plants at 96 HAT. The highest concentration of ^14^C-glyphosate is highlighted in red. Arrows indicate the treated leaf.

The differences in foliar uptake of ^14^C-glyphosate between the resistant hairy beggarticks populations compared to the S population were highly significant (*P* < 0.0001; DF = 2; *n* = 60) (**Figure 3A**). The amount of ^14^C-glyphosate absorbed ranged between 29.7 and 47.8%, 13.9 and 38.5%, 15.2 and 41.6%, for populations S, R1, and R2, respectively, between 24 and 96 HAT. At 24 and 96 HAT, the S population showed a greater uptake compared to R1 and R2 populations. However, after 48 and 72 HAT, the values were similar in the three populations.

With respect to the ^14^C-glyphosate translocation, the initial amount quantified from 68.2% at 24 HAT in the treated leaf diminished to 42.6% at 96 HAT in S population. Conversely, the larger amount of herbicide applied was retained in the leaf treated in the resistant populations, dropping from 79.6 to 64.6% in R1 population, and from 73.3 to 59.7% in R2 population at 24 and 96 HAT, respectively. In the S population, an average of 24.9% of the glyphosate translocated reached the root at 96 HAT, whereas in R1 and R2 populations it was only of 12.9 and 15.5%, respectively. **Table 2** shows the results of the percentage of ^14^C-glyphosate translocated to the remainder of the plant and root in hairy beggarticks plants.

**Table 2:** Translocation percentage of ^14^C-glyphosate in plants of glyphosate-susceptible and -resistant *Bidens pilosa* populations.

Population	HAT	Traslocation (% from uptake)*^a^*		
		Treated leaf	Remainder of plant	Root
S	24	68.2 ± 2.0 c	18.3 ± 1.1 ef	13.5 ± 1.0 cde
	48	59.7 ± 1.6 d	23.0 ± 1.6 c	18.5 ± 1.2 b
	72	52.3 ± 2.2 e	29.1 ± 1.7 b	19.6 ± 0.6 b
	96	42.6 ± 2.0 f	33.5 ± 0.6 a	24.9 ± 1.5 a
R1	24	79.6 ± 2.1 a	13.0 ± 0.5 g	7.4 ± 2.3 g
	48	73.6 ± 2.3 b	16.9 ± 3.2 ef	10.5 ± 0.9 f
	72	67.6 ± 3.3 c	19.1 ± 2.4 e	13.3 ± 1.3 cde
	96	64.6 ± 2.0 c	22.5 ± 2.5 cd	12.9 ± 1.0 de
R2	24	73.3 ± 2.3 b	15.2 ± 0.8 fg	11.5 ± 1.3 ef
	48	68.5 ± 1.9 b	17.8 ± 2.2 ef	13.7 ± 1.6 cde
	72	66.2 ± 4.1 c	19.4 ± 1.9 de	14.4 ± 1.2 cd
	96	59.7 ± 2.8 d	24.8 ± 2.3 c	15.5 ± 1.3 c

**^a^*Means with different letter within a column are statistically different at 95% probability determined by the Tukey test. ±Standard error of the mean (*n* = 5).*

The images obtained in the Phosphor Imager confirmed the previous results obtained for translocation. At 96 HAT it was seen how, in the plants of resistant populations, the glyphosate was retained mainly in the treated leaf, and only small amounts were translocated across the remainder of the plant in comparison to the S population (**Figure 3B**).

### Metabolism

Glyphosate and its metabolites were quantified by reversed-polarity capillary-electrophoresis. There were no significant differences between hairy beggarticks populations. The amount of glyphosate quantified was of 100% at 4 DAT, and higher than 95% at 8 and 12 DAT from total applied. Only small amounts of AMPA and glyoxylate were detected at this time in all populations.

### Enzyme Activity

**FIGURE 4:**
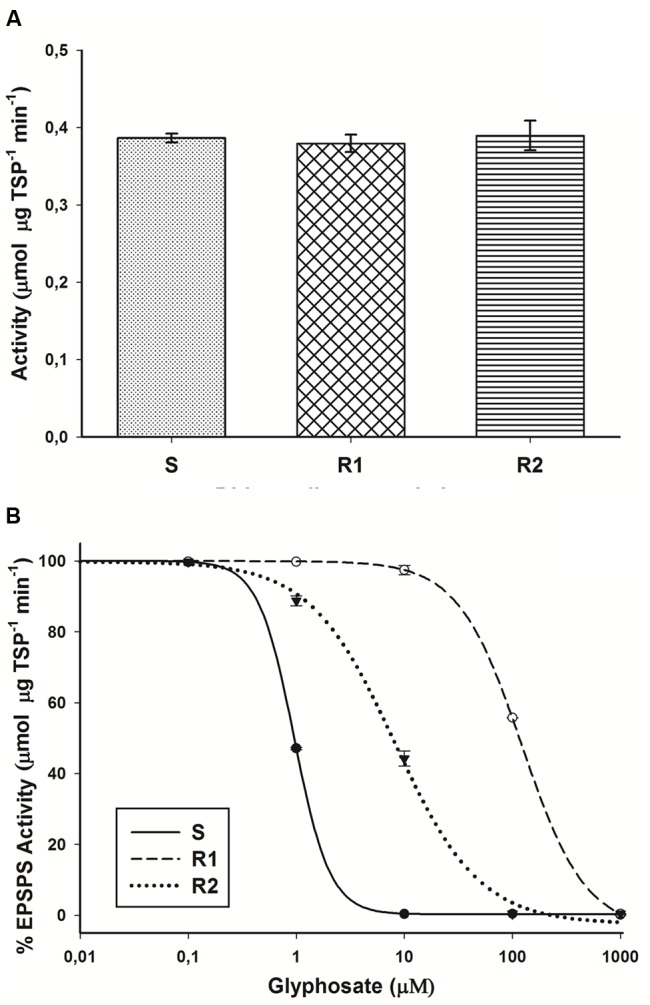
**5-enolpyruvyl shikimate-3-phosphate synthase (EPSPS) activity in glyphosate-susceptible and -resistant plants of *B. pilosa* populations. (A)** Basal EPSPS activity for glyphosate-susceptible and -resistant *B. pilosa* plants. Histograms represent treatment means and vertical bars SE of the mean (*n* = 3). **(B)** EPSPS enzyme activity expressed as percentage of the untreated control in leaf extracts of plants from glyphosate-susceptible and resistant *B. pilosa* populations. The equations of log–logistic curves to estimates the *I*_50_ values are: S: *Y* = 0.227 + {(99.98 - 0.227)/[1 + (dose/*I*_50_)^2.619^]}; R1: *Y* = -4.065 + {(99.92 + 4.065)/[1 + (dose/*I*_50_)^1.477^]}; R2: *Y* = -2.471 + {(99.79 + 2.471)/[1 + (dose/I_50_)^1.108^]}. Vertical bars represent the standard error of the mean (*n* = 3).

There were no significant differences (*P* = 0.34; DF = 2; *n* = 9) in the basal EPSPS activity (average = 0.39 μmol μg TSP^-1^ min^-1^) in plants of glyphosate-susceptible and -resistant hairy beggarticks populations in the absence of glyphosate (**Figure 4A**). The EPSPS enzyme was inhibited by glyphosate in plants of susceptible and resistant populations. For the S population, only 0.95 μM of glyphosate was necessary to inhibit EPSPS activity by 50% (*I*_50_). The resistant plants of R2 and R1 populations, on average, were 8.5- and 128.4-fold, respectively, less sensitive to glyphosate than the susceptible plants (**Figure 4B**).

### Sequencing of the EPSPS Gene

**FIGURE 5:**
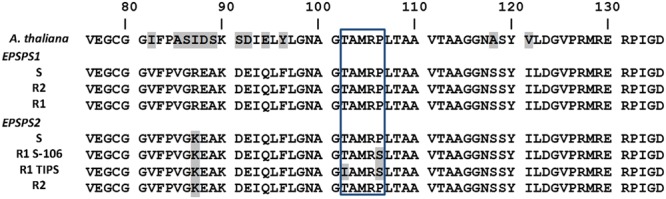
**Partial alignment of protein sequences of EPSPS1 and EPSPS2 genes in glyphosate-susceptible and -resistant *B. pilosa* populations.** The highlighted color indicates changes to codons from the consensus nucleotide sequence. Box includes from the 102 to 106 positions (amino acid number based on the start codon (ATG) of *A. thaliana* [GenBank: CAA29828.1] EPSPS sequence). The *B. pilosa* EPSPS cDNA sequences information can be found in GenBank with accession numbers KU984452–KU984458.

The sequencing from cDNA revealing the presence of two different EPSPS genes that are expressed in the three hairy beggarticks populations (**Table 3**; **Figure 5**), showed one homology above 92% between EPSPS1 and EPSPS2 genes based on their predicted proteins, and above 80% with respect to *Arabidopsis thaliana* (GenBank: CAA29828.1) (**Figure 5**). In the three populations, some individuals only showed the EPSPS1 gene, others the EPSPS2 gene, and others showed both genes.

**Table 3:** Frequency percentage of 5-enolpyruvyl shikimate-3-phosphate synthase (EPSPS) genes, and polymorphisms at 102 and 106 positions in glyphosate-susceptible and -resistant plants of *Bidens pilosa* populations.

Population	Number of individuals/clones	Gene	Gene Frequency (%)	Alleles^a^	Allele frequency (%)
S	5/15	EPSPS1	60.0	T102-P106	60.0
		EPSPS2	40.0	T102-P106	40.0
R1	20/60	EPSPS1	56.7	T102-P106	56.7
		EPSPS2	43.3	T102-P106	14.3
				T102-S106	23.3
				I102-S106	6.6
R2	5/15	EPSPS1	53.3	T102-P106	53.3
		EPSPS2	46.7	T102-P106	46.7

*^*a*^T102-P106 = wild type or glyphosate susceptible; T102-S106 = low-intermediate glyphosate resistance; and I102-S106 = high glyphosate resistance.*

Some R1 population plants were identified with a single mutation in Pro-106 position alone, and other plants presented a double mutation in the Thr-102 and Pro-106 positions in the EPSPS2 gene (**Figure 5**). The amino acid substitutions consisted of Threonine (ACC) to Isoleucine (ATC) in Thr-102 position, and from Proline (CCA) to Serine (TCA) in Pro-106 positon (**Figure 5**). Mutations were not found in the EPSPS1, and the R2 population did not show any mutation. Of the 20 individuals sequenced from R1 population, only 2 had the I102-S106 (TIPS) allele; 11 individuals the T102-S106 allele, and 7 had the wild type allele T102-P106 corresponding to 10, 55 and 35% of the sample size analyzed. Percentage frequency of EPSPS genes and alleles implicated in glyphosate resistance are shown in the **Table 3**.

## Discussion

The ED_50_, LD_50_ and *I*_50_ parameters (**Table 1**) showed the highest level of resistance developed by resistant hairy beggarticks populations. Our results of dose-response results are according with other glyphosate-resistant species. For example, the resistance index (RI) in resistant *C. bonariensis, C. canadensis*, and *C. sumatrensis* populations, asteraceae species like hairy beggarticks, ranged between 7- and 17-fold more compared to their respective susceptible population (Koger et al., 2004; González-Torralva et al., 2010, 2012b). Other dicotyledonous species such as *A. palmeri* and *Kochia scoparia* presented similar variation of resistance level showed RI between 3- and 18-fold (Whitaker et al., 2013; Ribeiro et al., 2014; Wiersma et al., 2015). A resistant *A. palmeri* population showed an RI 18-fold higher than its S population with an ED_50_ of 2565 g ae ha^-1^ (Whitaker et al., 2013).

On the basis of LD_50_, the RI ranged between 3- and 15-fold in weed species, such as *Lolium perenne* spp. *multiflorum* and *K. scoparia* (Salas et al., 2015; Wiersma et al., 2015). To achieve a total control in resistant hairy beggarticks populations, one needs to apply at least double the rate of glyphosate of that of their corresponding LD_50_. However, higher doses increase selection pressure and will accelerate the evolution of resistant populations (Salas et al., 2015).

Shikimic acid and enzyme EPSPS activity tests are accepted as appropriate parameters to determine susceptibility level to glyphosate (Dayan et al., 2015). With respect to EPSPS activity, some resistant *L. perenne* spp. *multiflorum* and *Echinochloa colona* populations showed differences in basal EPSPS activity compared to their respective susceptible populations (Salas et al., 2012; Alarcón-Reverte et al., 2015), but these differences were associated with a greater number of copies of the EPSPS gene. Therefore, the similar basal EPSPS activity between hairy beggarticks populations suggests that resistant populations could not have any differences in the number of copies of the EPSPS gene respect to susceptible populations. Multiple EPSPS copy numbers and/or increased EPSPS expression have been described as glyphosate resistance mechanisms in dicotyledonous species such as *A. palmeri, A. tuberculatus, K. scoparia* (Ribeiro et al., 2014; Chatham et al., 2015; Wiersma et al., 2015), among others species. However, similar gene copy numbers may not necessarily show the same level of resistance to glyphosate (Salas et al., 2012). As in resistant hairy beggarticks populations, higher glyphosate concentrations were necessary in resistant *L. perenne* spp. *multiflorum* and *E. colona* populations to inhibit EPSPS activity by 50% (*I*_50_) (Salas et al., 2012; Alarcón-Reverte et al., 2015).

In both assays of shikimic acid, the resistant hairy beggarticks populations showed a lesser shikimic acid accumulation than the S population (**Figure 2**). This evidenced the high susceptibility to glyphosate of the S population, and a different resistance level between R1 and R2 populations. Similar results have been reported in other species of glyphosate-resistant weeds, for instance, resistant *L. rigidum, E. colona*, and *Poa annua*) populations (Perez-Jones et al., 2007; Alarcón-Reverte et al., 2013; Cross et al., 2015). Any species with a low accumulation of shikimic acid requires a larger amount of glyphosate in order for it to be lethal (Cruz-Hipolito et al., 2011; Alcántara-de la Cruz et al., 2016). This can happen when, in the differential accumulation of shikimic acid, glyphosate does not reach the target site in sufficient amounts due to altered translocation patterns (Alarcón-Reverte et al., 2015; Cross et al., 2015). In this work, both susceptible and resistant populations accumulated shikimic acid. This indicated that the glyphosate arrived at the target site inhibiting the EPSPS, but that that inhibition was at different levels (Duke and Powles, 2008; Alarcón-Reverte et al., 2015), this being significantly greater in the S population.

Herbicide foliar retention and uptake are influenced by physiological and morphological traits (Cruz-Hipolito et al., 2011; Alcántara-de la Cruz et al., 2016), and are not major mechanisms conferring glyphosate resistance. Foliar retention capacity depends on the phenology of the plants. Studies on *Conyza* spp. showed that foliar retention was greater during the elongation of the stem than during flowering (González-Torralva et al., 2010). Only few weed species have presented differences in reduced glyphosate uptake and foliar retention as mechanisms involved in their resistance. For instance, resistant *A. tuberculatus, L. multiflorum*, and *Digitaria insularis* populations showed a reduced uptake (Michitte et al., 2007; de Carvalho et al., 2012; Nandula et al., 2013); and only *L. multiflorum* presented a lower foliar retention. These traits play an important role in innate glyphosate-tolerant species (Cruz-Hipolito et al., 2011; Alcántara-de la Cruz et al., 2016). In hairy beggarticks populations neither foliar retention nor ^14^C-glyphosate uptake were not mechanisms involved in the resistance. However, the ^14^C-glyphosate translocation results suggest that a reduced translocation could be the main mechanism involved in resistance (**Table 2**; **Figure 3B**), but the different resistance levels and shikimic acid accumulation between resistant hairy beggarticks populations suggests that their resistance mechanisms may differ between each other. Sammons and Gaines (2014) pointed out several cases with at least two resistance mechanisms. These cases involved TSR and NTSR mechanisms.

Shikimic acid pathway consists at least of two separate pathways, the presence of multiple EPSPS genes or isoforms is common in higher plants (Jensen, 1986). EPSPS isozymes have been identified differentially localized in plastids and cytosol (Mousdale and Coggings, 1985). This justifies the presence of the two EPSPS genes identified in hairy beggarticks populations. Weed species such as *C. sumatrensis* and *E. colona* also expressed two genes of EPSPS, one of them showing a mutation in Pro-106 position conferring glyphosate resistance (González-Torralva et al., 2014; Alarcón-Reverte et al., 2015).

A single Pro-106 mutation has been widely described in several resistant weed species to glyphosate (González-Torralva et al., 2012a, 2014; Alarcón-Reverte et al., 2015; Cross et al., 2015; Salas et al., 2015; between others). The levels of resistance conferred by single target-site mutation to date tend to vary at between 2- and 10-fold, depending on whether a target site mutation or reduced translocation is the mechanism (Bostamam et al., 2012), and up to 4 to 15-fold when two different mechanisms are involved (Sammons and Gaines, 2014), for instance, resistant *A. tuberculatus* (Nandula et al., 2013), *L. rigidum* (Yu et al., 2007; Bostamam et al., 2012) and *L. multiflorum* (González-Torralva et al., 2012a) populations, exhibited a mutation in Pro-106 position, and a reduced translocation with an RI fivefold for the resistant *A. tuberculatus* and *L. multiflorum* populations, whereas for those of *L. rigidum*, the RI was six–eightfold (Bostamam et al., 2012), and 14-fold (Yu et al., 2007) higher than their susceptible population.

The Thr-102-Ile mutation would be unlikely to occur first or independently (Weinreich et al., 2006), it has usually been associated with the Pro-106-Ser mutation, commonly known as TIPS. Such mutation has been reported to provide high resistance/tolerance to glyphosate in studies with *E. coli* (Healy-Fried et al., 2007), and it is used in transgenic glyphosate-resistant crops (see **Table 3**, review by Sammons and Gaines, 2014). Recently, the TIPS mutation was identified in two resistant *E. indica* populations. The latter was one population from Malaysia, as the first TIPS mutation naturally identified in weed species (Yu et al., 2015), and one from China (Chen et al., 2015). In both cases, the frequency of I102-S106 (high resistance) allele corresponding to TIPS mutation was lower (26%) of frequency in the population from the Malaysia (3 from 193 individuals), and 16.7% in the population from China (8 from 30 individuals) than T102-S106 (low-intermediate resistance) and/or T102-P106 (wild type or susceptible) alleles. The hairy beggarticks R1 population also presented a low frequency (6.6%) of I102-S106 allele in 2 from 20 individuals studied. This is another example of naturally evolved of TIPS mutation. Due to the insect pollination that shows hairy beggarticks, the allele of TIPS mutation could be easily spread to other Persian lime groves by insects. For this reason, future studies will focus on characterizing glyphosate-resistant genotypes using dCAPS markers, as well as the fitness cost of glyphosate resistance in this species.

Glyphosate-resistant species such as *D. insularis* and *C. canadensis* (de Carvalho et al., 2012; González-Torralva et al., 2012b) presented metabolism as a mechanism of resistance. However, the metabolism was not involved in the resistance to glyphosate in hairy beggarticks populations of this study. These results were consistent with other studies in *C. canadensis* and *E. colona* (Dinelli et al., 2006; Alarcón-Reverte et al., 2015), which demonstrated no contribution to resistance.

Additionally, EPSPS isozymes may have different response to glyphosate (Ream et al., 1988). Due to each isozyme was not isolated individually, we do not know which isozyme (EPSPS1- or EPSPS2-isozyme) is more sensitive to glyphosate. This can explain the differences observed in the EPSPS activity between the S and R2 populations, were no mutation was identified.

## Conclusion

These results revealed the first case of a double mutation (TIPS) evolved on the target site in a wild dicotyledonous weed, due to high selection pressure exerted by repeated glyphosate applications (3–4 times per year) in citrus groves from Mexico (Pérez-López et al., 2014).

The reduced translocation and the relationship between the parameters of ED_50_, LD_50_, and *I*_50_ confirmed glyphosate resistance of hairy-beggarticks. The R2 population used reduced translocation of ^14^C-glyphosate, to its target site (EPSPS) as major mechanism, to resist against glyphosate presenting a low-intermediate resistance. Although Ser-106 and TIPS mutations found in the EPSPS2 gene presented a low frequency, in association with a reduced glyphosate translocation, those were responsible for conferring high resistance in the R1 population.

The confirmation of this resistance suggests the need to include other pre-emergent and post-emergent herbicides to manage hairy beggarticks in citrus groves. In addition, practices that contributing to the germination and presence of susceptible hairy beggarticks plants, allowing their reproduction with resistant plants in order to reduce the resistance level.

## Author Contributions

JD-V, Provided the seeds used in this work. RA, HC-H, FB, JD-V, and RP, Idea and designed the experiments. RA, AR-D, CO, and PF-M, Performed the research. RA, AR-D, CO, and PF-M. Interpretation and analysis of results (of raw data). RA, AR-D, CO, HC-H, FB, JD-V, and RP: Wrote and approved the manuscript.

## Conflict of Interest Statement

The authors declare that the research was conducted in the absence of any commercial or financial relationships that could be construed as a potential conflict of interest.

## Footnotes

1 http://www.bioinformatics.nl/cgi-bin/primer3plus/primer3plus.cgi
